# Transmembrane protein TMEM170A is a newly discovered regulator of ER and nuclear envelope morphogenesis in human cells

**DOI:** 10.1242/jcs.175273

**Published:** 2016-04-15

**Authors:** Andri Christodoulou, Rachel Santarella-Mellwig, Niovi Santama, Iain W. Mattaj

**Affiliations:** 1 Department of Biological Sciences, University of Cyprus, Nicosia, Cyprus; 2 European Molecular Biology Laboratory, Heidelberg, Germany

**Keywords:** TMEM170A, Reticulon, Endoplasmic reticulum, Nuclear envelope, Nuclear pore complex

## Abstract

The mechanism of endoplasmic reticulum (ER) morphogenesis is incompletely understood. ER tubules are shaped by the reticulons (RTNs) and DP1/Yop1p family members, but the mechanism of ER sheet formation is much less clear. Here, we characterize TMEM170A, a human transmembrane protein, which localizes in ER and nuclear envelope membranes. Silencing or overexpressing TMEM170A in HeLa K cells alters ER shape and morphology. Ultrastructural analysis reveals that downregulation of TMEM170A specifically induces tubular ER formation, whereas overexpression of TMEM170A induces ER sheet formation, indicating that TMEM170A is a newly discovered ER-sheet-promoting protein. Additionally, downregulation of TMEM170A alters nuclear shape and size, decreases the density of nuclear pore complexes (NPCs) in the nuclear envelope and causes either a reduction in inner nuclear membrane (INM) proteins or their relocalization to the ER. TMEM170A interacts with RTN4, a member of the reticulon family; simultaneous co-silencing of TMEM170A and RTN4 rescues ER, NPC and nuclear-envelope-related phenotypes, implying that the two proteins have antagonistic effects on ER membrane organization, and nuclear envelope and NPC formation.

## Abstract (precis)

**Highlighted Article:** TMEM170A is a human ER and nuclear envelope transmembrane protein. Down- and overexpression induce tubular or sheet ER formation, respectively, indicating that TMEM170A is a newly discovered ER-sheet-promoting protein.

## INTRODUCTION

The endoplasmic reticulum (ER) is a continuous membranous system consisting of the nuclear envelope and the peripheral ER, a network of interconnected tubules and flat sheets (Baumann and Walz, 2001; Lynes and Simmen, 2011). Each part of the ER has a distinct morphology and is involved in different cellular processes (Baumann and Walz, 2001; Shibata et al., 2006; Hu et al., 2011; Lin et al., 2012; Chen et al., 2013; Goyal and Blackstone, 2013). How the ER structure is formed and maintained is still not fully understood.

The morphology of tubular ER is regulated by reticulon (RTN) and DP1/Yop1p family members. The reticulon and DP1/Yop1p families are not related by sequence homology but they each contain two long hydrophobic transmembrane domains that form a wedge-like structure within the outer leaflet of the lipid bilayer, thereby generating a positive curvature in the ER membrane (Voeltz et al., 2006; Hu et al., 2008; Shibata et al., 2008). The reticulons and DP1/Yop1p also form homo- and hetero-oligomers that serve as arc-like scaffolds around a membrane tubule and stabilize it (Shibata et al., 2008). Fusion of ER tubules, which is mediated by atlastin, an evolutionarily conserved dynamin-like GTPase, leads to the formation of three-way tubular junctions and the generation of a polygonal network (Hu et al., 2009; Orso et al., 2009; Moss et al., 2011).

How flat ER sheets are shaped is less well understood. The reticulon and DP1/Yop1p family members localize at the edges of the flat sheets and play a role in ER sheet formation (Shibata et al., 2010). In addition, proteins of the translocon complex and coiled-coil transmembrane proteins such as CLIMP-63 (also known as CKAP4), kinectin and p180 (also known as RRBP1) can regulate ER sheet formation and stabilize the flatness of the sheets (Shibata et al., 2006, 2010; Puhka et al., 2007). The coiled-coil domain of CLIMP-63 sits within the ER lumen and forms intraluminal bridges that maintain the constant luminal thickness of ER sheets (Klopfenstein et al., 2001).

The nuclear envelope is composed of two lipid bilayers, the outer nuclear membrane (ONM), which is contiguous with the peripheral ER, and the inner nuclear membrane (INM), which contains a unique set of proteins that localize by means of direct or indirect contacts with the nuclear lamina and/or chromatin. The two membranes form flat membrane sheet-like structures, which are fused at the nuclear pore complexes (NPC) through which selective bidirectional transport of macromolecules occurs (Hetzer, 2010; Wente and Rout, 2010).

The ER is not a static structure but undergoes dramatic changes during the cell cycle. At the beginning of mitosis, the nuclear envelope disassembles and its membrane components become part of the mitotic ER (Ellenberg et al., 1997; Yang et al., 1997). At the end of mitosis, the nuclear envelope re-emerges from the mitotic ER and is reformed around the segregated chromosomes. The mechanism through which the segregation of nuclear envelope membranes occurs is still a matter of debate. One model proposes that during mitosis, ER tubules transform into sheets (Poteryaev et al., 2005; Lu et al., 2009, 2011) and that, at the end of mitosis, nuclear envelope assembly is initiated by ER sheets that contact the chromatin (Lu et al., 2011). The second model proposes that ER sheets are transformed into fenestrated sheets and tubules late in mitosis (Anderson and Hetzer, 2007; Puhka et al., 2007, 2012; Wang et al., 2013) and that the ends of ER tubules first contact chromatin, then flatten into membrane sheets that spread around the chromatin. Despite the diverse views on how the nuclear envelope is formed, it is well-understood that membrane-shaping proteins, especially those affecting the transition of tubular ER to ER sheets, are key players in nuclear envelope formation. For example, depletion of reticulon proteins (RTN1, RTN3, RTN4) by RNA interference promotes ER sheet proliferation and accelerates nuclear envelope formation, whereas overexpression of RTNs promotes tubular ER proliferation and results in a delay in nuclear envelope formation (Anderson and Hetzer, 2008). The discovery of ER-sheet-shaping proteins is an important step towards a full understanding of how the peripheral ER and the nuclear envelope are segregated and maintained.

In this work we identify and characterize TMEM170A, showing that it is a newly discovered ER-sheet-promoting protein whose concentration not only affects peripheral ER structure but also influences nuclear envelope expansion, NPC formation and INM protein targeting. In addition, we report that TMEM170A interacts with RTN4 and demonstrate that these two proteins act antagonistically on ER shape, and nuclear envelope and NPC formation.

## RESULTS

### Characterization of human TMEM170A protein

**Fig. 1.**
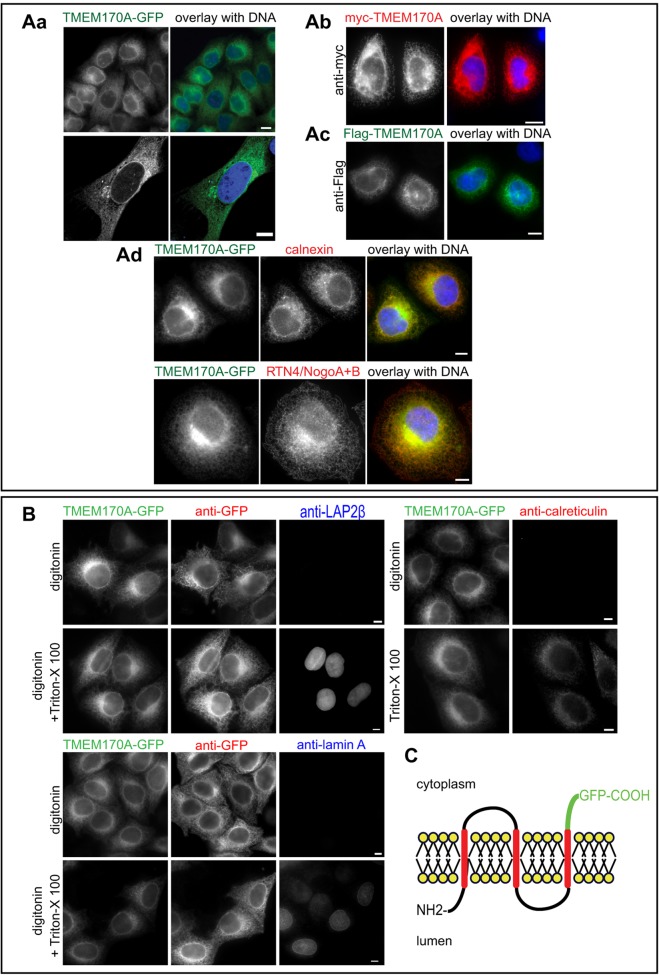
**Localization and membrane topology of human TMEM170A.** (A) TMEM170A localizes to the nuclear envelope and the ER. (Aa–c) Immunofluorescence of HeLa K cells transiently transfected with TMEM170A–GFP (green), FLAG–TMEM170A (green) or myc–TMEM170A (red). DNA was visualized with Hoechst in all panels (blue). (Ad) Immunofluorescence of HeLa K cells transiently transfected with TMEM170A–GFP (green) and co-stained for ER markers calnexin (red) or RTN4/NogoA+B (red). (B) The C-terminal domain of TMEM170A faces the cytoplasm. HeLa K cells, stably expressing TMEM170A–GFP, were fixed and subjected to 0.05% w/v digitonin to permeabilize the plasma membrane or 0.05% w/v digitonin+0.5% v/v Triton X-100 to permeabilize both the plasma membrane and the nuclear envelope. Cells were stained with anti-GFP antibody (green) in combination with either anti-LAP2β or anti-lamin-A or calreticulin (red). TMEM170A–GFP is accessible to the anti-GFP antibody in digitonin-only semi-permeabilized cells, whereas LAP2β, lamin A and calreticulin are only recognized upon full permeabilization. (C) Schematic of the membrane topology of TMEM170A. Transmembrane segments were predicted with TMPRED and topology of the C-terminus was confirmed in the experiments shown in B that support the model shown. Scale bars: 10 μm.

The transmembrane protein TMEM170A is conserved in major eukaryotic phyla (Fig. S1) but is thus far functionally uncharacterized. We identified human TMEM170A (Q8WVE7, UniProtKB/Swiss-Prot) as an ER and nuclear envelope protein in human cells through initial analysis of the location of transiently transfected TMEM170A–GFP, FLAG–TMEM170A or myc–TMEM170A that, in all three cases, localized to both peripheral ER and nuclear envelope membranes (Fig. 1Aa–c). We proceeded to generate a stably transfected HeLa K cell line expressing TMEM170A fused with GFP at its C-terminus. As expected, TMEM170A–GFP was specifically located to ER and nuclear envelope membranes, extensively co-localized with the ER marker proteins calnexin and RNT4 (Fig. 1Ad).

Human TMEM170A is a small protein of 15.25 kDa containing three transmembrane domains whose membrane topology/orientation is unknown. Using the TMPRED software, the N-terminus is predicted to be luminal and the C-terminus cytoplasmic (Fig. 1C). In order to test this prediction, TMEM170A–GFP line cells were fixed and permeabilized either with digitonin alone or digitonin plus Triton X-100. Cells were then probed with anti-GFP antibody in combination with antibodies to lamin A (an INM protein), LAP2β (also an INM protein) or calreticulin (a resident luminal ER protein). We observed that the GFP tag was accessible to the anti-GFP antibody and gave signal in digitonin semi-permeabilized cells, whereas LAP2β, lamin A and calreticulin were, as expected, only accessible after digitonin plus Triton X-100 permeabilization (Fig. 1B). This indicated that the GFP moiety of TMEM170A–GFP is available to the antibody without the need for internal membrane solubilization by Triton X-100. We thus conclude that the C-terminus of TMEM170A is oriented toward the cytoplasm, as per the TMPRED prediction, and its N-terminus is, consequently, luminal (Fig. 1C).

### Downregulation of TMEM170A by RNAi alters ER shape and morphology

To start elucidating the biological role of TMEM170A in HeLa K cells, its expression was reduced by siRNA-mediated RNA interference (RNAi) and the effects on ER and nuclear envelope morphology were assessed. Two different sets of TMEM170A-specific siRNA oligonucleotides (set 2 and set 3, Table S3) were tested by real-time RT-PCR and immunofluorescence analysis. Both sets efficiently depleted TMEM170A expression and produced similar phenotypes after 72 h (set 2) and 48 h (set 3) of transfection; oligo set 2 was employed for further analysis (Fig. S2A–D).

**Fig. 2.**
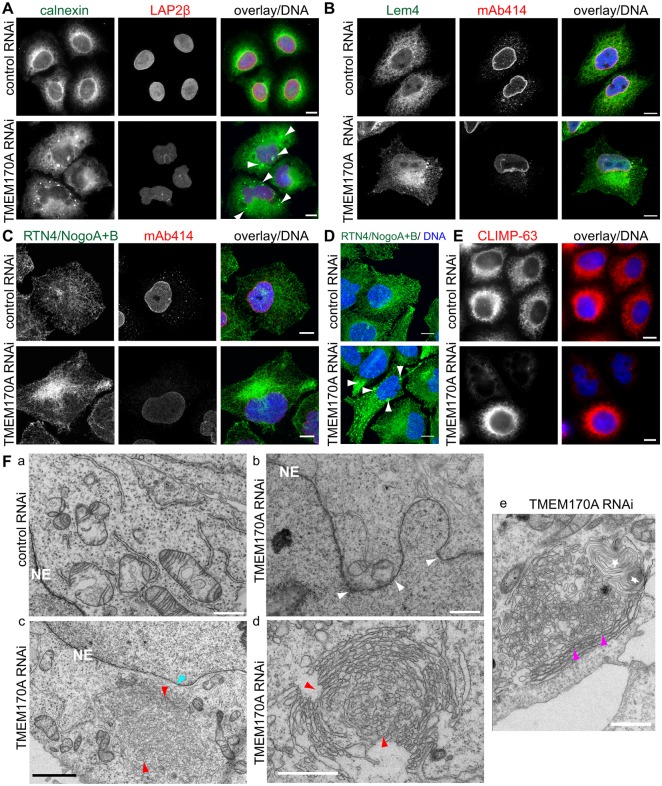
**Downregulation of TMEM170A alters ER structure and induces tubular ER proliferation.** (A–E) TMEM170A siRNA alters ER structure in HeLa K cells. Immunofluorescence analysis using antibodies to calnexin (A; green), LEM4 (B; green) or RTN4/NogoA+B (C,D; green) in combination with other nuclear envelope markers (as indicated; red) reveals that, in silenced cells, the ER is concentrated in a restricted area close to one side of the nucleus or is atypically widely spread and, additionally, ER aggregation is observed (white arrowheads; A,D) compared with controls. TMEM170A-silenced cells stained with anti-CLIMP-63 antibody (E; red) showed a reduced ER sheet signal, relative to control cells. Nuclei in blue. Scale bars: 10 μm. (F) TEM of the ER and nuclear envelope of control (Fa) and TMEM170A-silenced cells (Fb–e). TMEM170A silencing induces nuclear envelope invagination (white arrowheads, b) and increased tubular ER (red arrowheads, Fc; another example at higher magnification in Fd). Connections between nuclear envelope and the membranes were visible (Fc, light blue arrowhead). Occasionally, ER tubules were seen connected with part of semi-organized smooth ER (cisternae, purple arrowheads) and rarely whorls (white stars) (Fe). Scale bars: 1 μm (black) or 2 μm (white). Section thickness: 60 nm.

Downregulation of TMEM170A by siRNA caused considerable cell death. Compared with control cells, only 10±2% of silenced cells survived 72 h after transfection (Fig. S2E). Furthermore, surviving cells had noticeably altered ER morphology and irregularly shaped nuclei of enlarged size (Fig. 2A–D). TMEM170A-siRNA-treated cells were stained with antibodies for several ER proteins [calnexin, LEM4, RTN4 (also known as Nogo-A/B)] and compared with cells treated with negative control siRNA. In more than 80% of TMEM170A-silenced cells, the ER, as visualized by the antibodies to all three proteins, was atypical, often confined to a restricted area asymmetrically distributed around the nucleus or more distantly dispersed, and exhibited a propensity for aggregation when compared with its typically homogenous perinuclear localization in control cells (Fig. 2A–D). In addition, TMEM170A-silenced cells probed with anti-CLIMP-63 antibody, an ER-sheet-specific marker, showed reduced immunofluorescence signal compared with control cells (Fig. 2E; Fig. S2C3). Consistently, quantification by western blot showed a significant reduction of CLIMP-63 protein levels in TMEM170-silenced samples to 28.47±2.25% of control samples (*P*=6.89×10^−5^; Fig. S2D).

### Ultrastructural analysis reveals that downregulation of TMEM170A specifically induces tubular ER formation

The effects of TMEM170A knockdown on the morphology and organization of the ER were subsequently examined at higher resolution by transmission electron microscopy (TEM) and 3D electron tomography in HeLa K cells. TEM revealed that TMEM170A silencing induced frequent nuclear envelope in- or evagination, consistent with the observed altered shape of nuclei (Fig. 2F, arrowheads; compare control in Fig. 2Fa with silenced cell in Fig. 2Fb). The aggregates seen by light microscopy seem to be unorganized tubular ER in TEM, indicating that TMEM170A silencing induced tubular ER formation (Fig. 2Fc,d). Connections between nuclear envelope and the membranes were visible (Fig. 2Fc, light blue arrowhead). Occasionally, it was observed that aggregates consisted of tubular ER connected with part of semi-organized smooth ER (cisternae and rarely whorls; Fig. 2Fe). This observation probably indicated that, in these aggregates, tubule–tubule fusion occurred to some extent. Finally, there were occasions where aggregates appear to be, at first glance, well-organized structure of smooth ER but scanning through the 3D images revealed that the tubules were mostly still separated into small sections, rather than joined up into cisternal stacks (Movie 1). Well-organized smooth ER structures, such as those detected in response to elevated expression of resident ER proteins (Snapp et al., 2003), were not observed upon TMEM170A silencing.

Taking these results together, we conclude that downregulation of TMEM170A causes increased tubular ER formation that is mostly not organized. Occasional larger aggregates appear to be able to undergo fusion, to various extents, into more organized structures, but not into completely organized cisternae.

### Overexpression of TMEM170A induces ER sheet formation

**Fig. 3.**
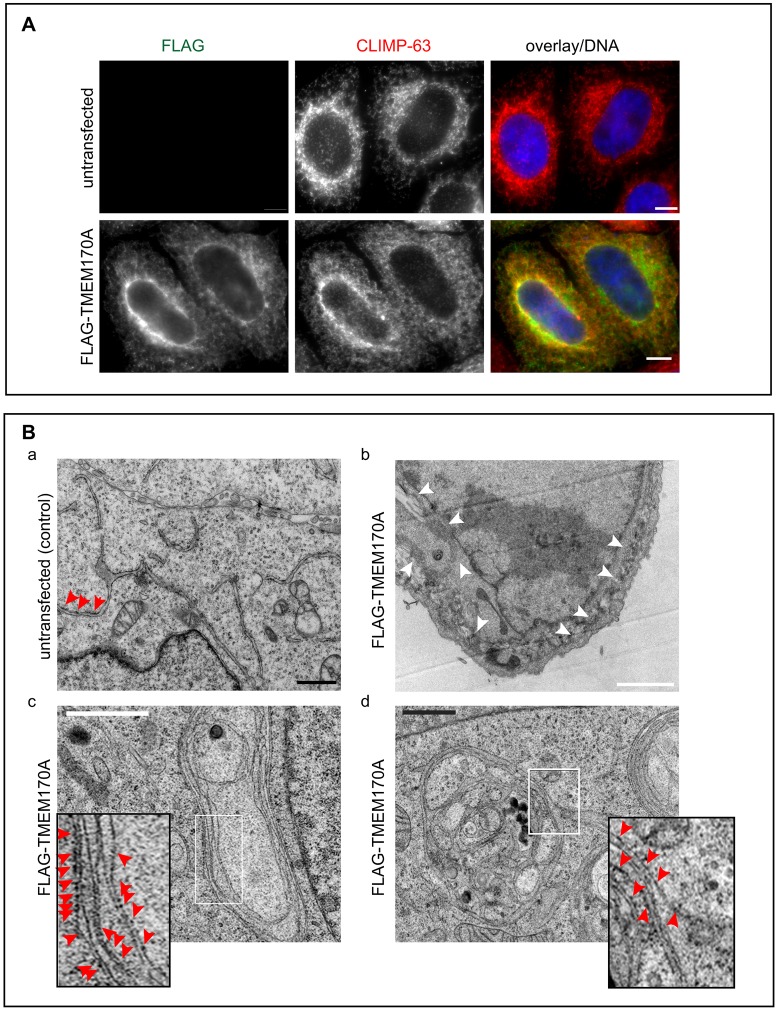
**Overexpression of TMEM170A induces ER sheet proliferation.** (A) Immunofluorescence of untransfected HeLa K cells or transiently transfected with FLAG–TMEM170A (green) in double labeling with ER sheet marker CLIMP-63 (red); nuclei (blue). Scale bars: 10 μm. (B) TEM images of HeLa K cells untransfected (Ba) and overexpressing FLAG–TMEM170A (Bb–d). Overexpressing cells have a highly enriched ER, composed of prominent well-organized and extensive ER sheet stacks with membrane-bound ribosomes (Bb–d) as compared with untransfected cells (Ba). Small images are a magnification of a representative region (boxed). Red arrowheads indicate ribosomes present on the ER sheets, white arrowheads in Bb indicate ER sheet stacks, which are extensive throughout the cell. Scale bars: 1 μm (black) or 2 μm (white). Section thickness 60 nm. In Ba,b cells were chemically fixed; in Bc,d they were high-pressure frozen.

We speculated that if downregulation of TMEM170A induces the formation of tubular ER, then its overexpression might induce proliferation of ER sheets. We first tested this by analyzing ER structure in FLAG–TMEM170A-overexpressing, transiently transfected HeLa K cells by indirect immunofluorescent microscopy. We observed that cells overexpressing TMEM170A developed an expanded volume of CLIMP-63-positive ER (Fig. 3A). Examining the ER structure at high resolution by TEM and 3D electron tomography in overexpressing cells revealed the presence of highly proliferated ER, composed of prominent well-organized and extensive ER sheet stacks, decorated with membrane-bound ribosomes (Fig. 3Bb–d; Movie 2; compare with control in Fig. 3Ba). Taken together with the silencing results, this indicated that the level of cellular TMEM170A protein appears to affect membrane morphogenesis in the ER and specifically the ratio between tubular ER and ER sheets.

### Downregulation of TMEM170A by siRNA alters nuclear shape and size, and decreases NPC density

As mentioned above, silencing of TMEM170A resulted in distinct nuclear envelope phenotypes, namely irregular shape and increased size, in addition to changes in the organization of ER membranes with which the nuclear envelope is connected. To investigate the nuclear events that were affected by downregulation of TMEM170A, we performed a series of experiments. We first measured both the nuclear surface area and nuclear volume to quantify their size in TMEM170A-silenced nuclei and compare it with control-silenced nuclei. We found that the nuclear surface area of TMEM170A-silenced cells was enlarged to 145.68±4.82% of the nuclear surface area of control-silenced cells (622.21±6.87 μm^2^ in controls vs 906.58±36.52 μm^2^ in silenced cells, *P*=0.00019; Fig. S2F). We also found that nuclear volume of TMEM170A-silenced cells was enlarged to 137.38±1.13% the nuclei of control-silenced cells (867.91±16.51 μm^3^ in controls vs 1192.4±28.39 μm^3^ in silenced cells, *P*=6.84×10^−5^; Fig. S2G). We speculated that if downregulation of TMEM170A causes enlargement of nuclear surface area and nuclear volume, then its overexpression might cause a reduction. Indeed, overexpression of FLAG–TMEM170A caused a reduction of nuclear surface area to 83.9±1.9% of control cells (622.21±6.87 μm^2^ in controls vs 522.14±15.88 μm^2^ in FLAG–TMEM170A-overexpressing cells, *P*=0.0005; Fig. S2F) and a reduction of nuclear volume to 73.97±19.82% of control cells (867.91±16.51 μm^3^ in controls vs 643.31±25.47 μm^3^ in FLAG–TMEM170A overexpressing cells, *P*=0.0002; Fig. S2G). Taking these results together, we conclude that TMEM170A is implicated in a mechanism that controls nuclear size or expansion.

**Fig. 4.**
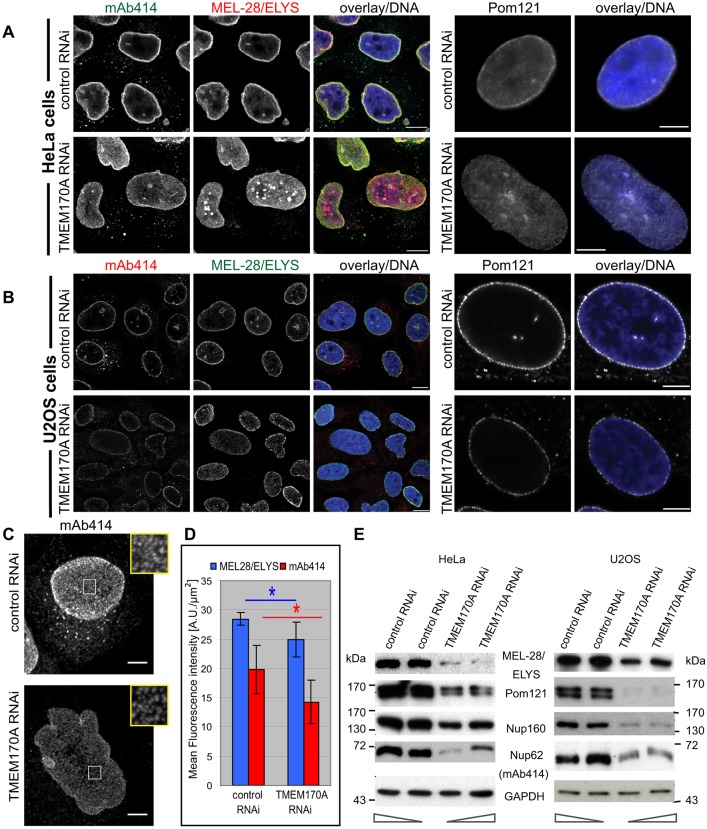
**Downregulation of TMEM170A by siRNA alters nuclear shape and size and causes a significant decrease in NPC numbers.** (A) siRNA knockdown of TMEM170A reduced nuclear rim signal in HeLa K cells. Cells were transfected with negative control or TMEM170A siRNAs and, at 72 h post-transfection, fixed and stained with mAb414 (green), anti-MEL28/ELYS (red) or anti-Pom121 (white) antibodies. Nuclei counterstained in blue in all panels. Scale bars: 10 μm. (B) TMEM170A siRNA also causes reduction of nuclear rim signal in U2OS cells. Control or TMEM170A-silenced U2OS cells were stained with mAb414 (red) and anti-MEL28/ELYS (green) or anti-Pom121 (white) antibodies. Scale bars: 10 μm (left panels) or 5 μm (right panels). (C) NPCs, at the bottom surface of the nuclear envelope of control- and TMEM170A-silenced HeLa K cells, were visualized by indirect immunofluorescence using mAb414 antibody. Representative areas of each cell are magnified in the boxes. Scale bars: 5 μm. (D) Quantitation of mean immunofluorescence of anti-MEL28/ELYS and mAb414 antibodies to estimate NPC density in HeLa K cells treated with control or TMEM170A siRNAs (*n*>52 nuclei per condition from three independent experiments). Error bars show s.d.; *P<0.05. TMEM170A-silenced cells displayed a reduction of anti-MEL28/ELYS antibody mean fluorescence intensity to 69.58±12.7% of the control-silenced cells (*P=*0.025), whereas TMEM170A-silenced cells showed a reduction of mAb414 mean fluorescence intensity to 58.72±22.16% of the control-silenced cells (*P=*0.018). (E) TMEM170A siRNA reduces protein levels of nucleoporins Nup62, Nup160, ELYS and Pom121 in HeLa K or U2OS total cell protein extracts from control and TMEM170A-silenced cells, as assayed by western blot. Two different amounts (1/5 and 1/7 of total lysate) from each protein extract were loaded on the gel, as indicated by arrows at the bottom.

We next investigated whether nuclear pore complex (NPC) formation was influenced by TMEM170A silencing, using several nucleoporin markers. TMEM170A-silenced cells, probed with antibody mAb414, which recognizes the FXFG repeats present in several nucleoporins (Nup62, Nup214, Nup358 and Nup153), exhibited reduced signal at the nuclear rim relative to control cells (Fig. 4A). We also observed reduced nuclear rim staining with antibodies against nucleoporins ELYS (also known as MEL28) and Pom121 (Fig. 4A). These results were replicated in another human cell line, osteosarcoma U2OS cells (Fig. 4B). To verify whether the overall number of pores is affected by TMEM170A knockdown, we acquired confocal images of HeLa K cells stained with anti-MEL28/ELYS and mAb414 antibodies and estimated pore density by measuring the mean fluorescence intensity of ELYS and mAb414, respectively, in each nucleus (Fig. 4C). We observed a reduction of NPC density in silenced cells to 69.58±12.7% of control cells, based on ELYS immunofluorescence (28.42±1.06 A.U./μm^2^ in controls vs 19.85±4.15 A.U./μm^2^ in silenced cells, *P*=0.025; Fig. 4D) and a reduction of NPC density in silenced cells to 58.72±22.16% compared with control cells, based on mAb414 immunofluorescence (24.93±2.96 A.U./μm^2^ in controls vs 14.26±3.76 A.U./μm^2^ in silenced cells, *P*=0.018; Fig. 4D). The reduction of mAb414 immunofluorescence is greater than that of ELYS, most probably because of the formation of ELYS aggregates (seen in Fig. 4A), contributing to higher mean signal intensity in this case. In addition to the decrease in the NPC density, cellular levels of various nucleoporins, including Nup62, Nup160, ELYS and Pom121 were reduced in both cell lines following silencing, as revealed by western blot analysis (Fig. 4E). Quantification of Nup62 protein levels by western blot showed a significant reduction of Nup62 protein levels in TMEM170-silenced samples to 29.49± 5.24% of control-silenced samples (*P=*0.0009; Fig. S2D). On the basis of these results, we conclude that reduction in TMEM170A reduces NPC formation or accumulation.

### TMEM170A silencing also causes depletion or mislocalization of INM proteins

**Fig. 5.**
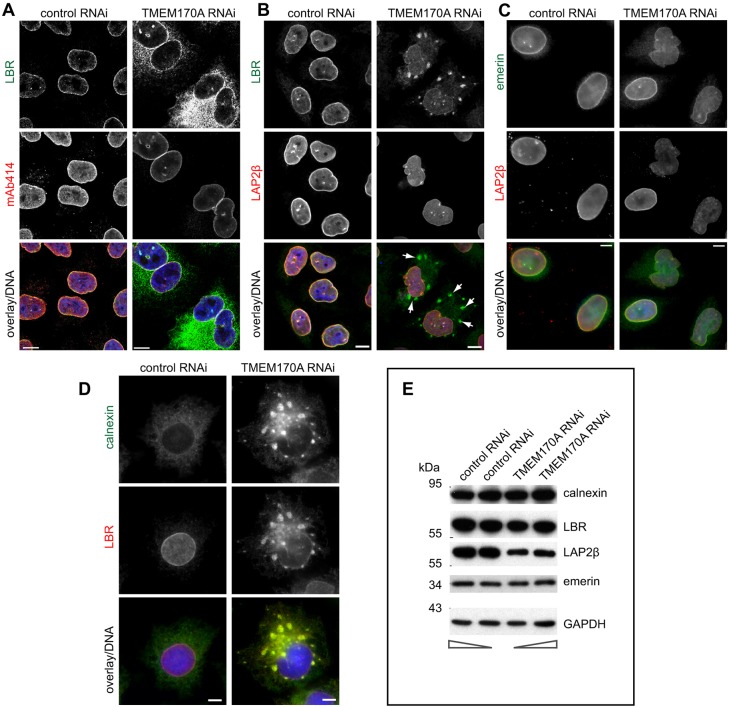
**TMEM170A silencing also causes depletion or mislocalization of INM proteins.** (A) TMEM170A silencing affects targeting of LBR to the INM. LBR (green) is strongly mislocalized to the ER in TMEM170A-silenced cells, compared with control cells. Triple staining with mAb414 (red) and nuclei in blue. Scale bars: 10 μm. (B) LBR (green) also localizes in aggregates in the ER (arrowheads) in most of the silenced cells, unlike in control cells. Silenced cells, stained with anti-LAP2β antibody (red), show reduced signal at the nuclear rim. Nuclei in blue. Scale bars: 10 μm. (C) Emerin (green) nuclear rim staining was reduced in TMEM170A-silenced cells, compared with controls. LAP2β (red), as in B, shows a reduced nuclear rim signal in silenced cells. Nuclei in blue. Scale bars: 10 μm. (D) LBR (red) colocalizes with calnexin aggregates (green) in the ER of TMEM170A-silenced cells. Scale bars: 10 μm. (E) Total HeLa K cell extracts from control and TMEM170A-silenced cells were assayed by western blot. The levels of LBR, emerin and calnexin were unaffected but those of LAP2β were specifically reduced in silenced cells. Two different amounts (1/5 and 1/7 of total lysate) of each protein extract were loaded on the gel, as indicated by arrows (bottom).

To further probe the observed reduction in nucleoporins and NPCs in the absence of TMEM170A, we examined the distribution of several INM proteins. Control and TMEM170A-silenced cells were stained with antibodies against LAP2β, LBR and emerin. TMEM170A-silenced cells stained with anti-LAP2β antibody showed a reduced signal at the nuclear rim (Fig. 5B,C; Fig. S2A1). Emerin nuclear rim staining was also reduced and, in some RNAi-treated cells, partial mislocalization from the INM to the ER was visible (Fig. 5C). LBR was prominently mislocalized to the ER in more than 80% of the silenced cells and also found in striking ER-associated aggregates in nearly 20% of the cells (Fig. 5A,B; Fig. S2C3). These LBR-containing ER aggregates also contained calnexin (Fig. 5D). The amount of LAP2β was reduced by TMEM170A RNAi, whereas there was no change in the levels of emerin or LBR (Fig. 5E). Quantification of LAP2β and calnexin protein levels by western blot showed a reduction of LAP2β to 30.73±12.42% of control (*P=*0.02) and no reduction of calnexin protein levels in TMEM170-silenced, compared with control-silenced samples (Fig. S2D).

### Rescue experiments

**Fig. 6.**
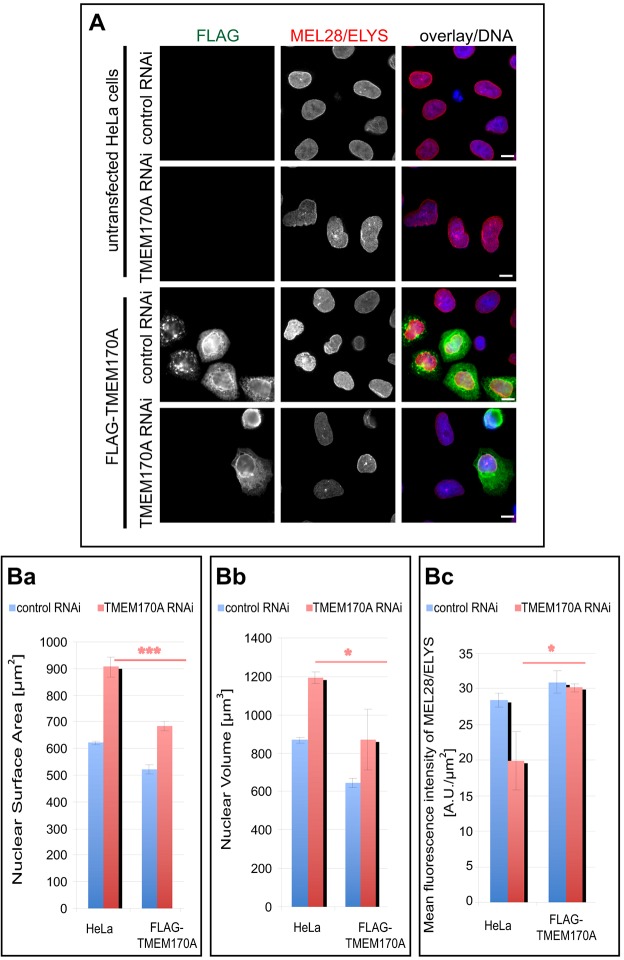
**FLAG–TMEM170A expression rescues the phenotype caused by TMEM170A silencing in HeLa K cells.** (A) HeLa K cells were transfected with control or TMEM170A siRNAs and, 48 h later, with a plasmid expressing FLAG–TMEM170A. Cells were fixed after a further 24 h and stained with anti-MEL28/ELYS (red) and anti-FLAG (green) antibodies. Nuclei in blue. Scale bars: 10 μm. (B) Quantitation of nuclear size (Ba), nuclear volume (Bb), and mean nuclear immunofluorescence of anti-MEL28/ELYS antibody signal to estimate the NPC density (Bc) of cells silenced with control or TMEM170A siRNAi and transiently transfected or not with FLAG–TMEM170A [HeLa K+ control RNAi (*n*=52 cells), HeLa K+ TMEM170A RNAi (*n*=52), FLAG–TMEM170A+control RNAi (*n*=52) and FLAG–TMEM170A+TMEM170A RNAi (*n*=34)]. For each parameter, the average of three independent experiments per condition is displayed; error bars show s.d.; *P<0.05, ***P<0.001. In all cases, FLAG–TMEM170A+TMEM170A RNAi restored the phenotype caused by single TMEM170A silencing in a statistically significant manner.

In order to further confirm the specificity of the silencing phenotypes we observed (also Fig. S2A–D), we carried out a series of rescue experiments by transfecting control- or TMEM170A-silenced cells with a construct expressing FLAG-tagged TMEM170A, which should be expected to restore TMEM170A protein levels, given that our siRNA oligo #2 targets the 3′UTR of TMEM170A mRNA (Fig. 6).

We first investigated if expression of FLAG–TMEM170A could rescue the altered ER shape/morphology observed upon TMEM170A silencing. As mentioned already in previous Results sections, in more than 80% of TMEM170A-silenced HeLa K cells the ER was confined to a restricted area asymmetrically distributed around the nucleus and with a propensity for aggregation, whereas more than 90% of overexpressing FLAG–TMEM170A cells showed a markedly expanded volume of ER sheets compared with control cells. The fact that the levels of cellular TMEM170A protein appears to affect the ratio between tubular ER and ER sheets in human cells therefore made this rescue experiment hard to execute. In rescue experiments, we observed that rescue of ER structure in TMEM170A-silenced cells by concurrent expression of FLAG–TMEM170A was modest, representing only a 10% increase in the number of cells displaying normal ER structure, compared with non-transfected, silenced cells (data not shown). Notably, the level of FLAG–TMEM170A expression in these rescued cells (as judged by the intensity of signal by anti-FLAG immunofluorescence) was moderate and not high.

We next investigated whether FLAG–TMEM170A expression could rescue the nuclear envelope phenotypes observed upon TMEM170A silencing. As already observed, TMEM170A-silenced HeLa K cells showed an enlargement of the nuclear surface area of TMEM170A-silenced cells to 145.68±4.82% of the nuclear surface area of control-silenced cells; however, in TMEM170A-silenced cells expressing FLAG–TMEM170A, nuclear surface area was now found to be considerably reduced (906.58±36.52 μm^2^ in TMEM170A-silenced cells vs 684.16±17.37 μm^2^ in TMEM170A-silenced cells expressing FLAG–TMEM170A, *P=*0.0006; nuclear surface area of control-silenced cells was 622.21±6.87 μm^2^; Fig. 6A,Ba). This indicated that FLAG–TMEM170A expression could rescue the nuclear surface area phenotype observed upon depletion of TMEM170A.

We next investigated whether FLAG–TMEM170A expression could also rescue the enlarged nuclear volume phenotypes. As already observed, TMEM170A-silenced cells showed an increase in nuclear volume to 137.38±1.13% of control-silenced cells; however, in TMEM170A-silenced cells expressing FLAG–TMEM170A nuclear volume was considerably reduced (1192.4±28.39 μm^3^ in TMEM170A-silenced cells vs 870.02±155.64 μm^3^ in TMEM170A-silenced cells expressing FLAG–TMEM170A, *P=*0.02; nuclear volume of control-silenced cells was 867.91±16.51 μm^3^; Fig. 6A,Bb). Again, this indicated that FLAG–TMEM170A expression could rescue the nuclear volume phenotype observed upon depletion of TMEM170A.

Finally, we tested if FLAG–TMEM170A expression could rescue the NPC phenotypes observed upon TMEM170A silencing. Specifically, TMEM170A-silenced cells showed a reduction of NPC density to 69.58±12.7% of control cells; expressing the FLAG–TMEM170A in TMEM170A-silenced cells, however, rescued this reduction (19.85±4.15 A.U./μm^2^ in TMEM170A-silenced cells vs 30.16±0.59 A.U./μm^2^ in TMEM170A-silenced cells expressing FLAG–TMEM170A, *P=*0.013; mean ELYS fluorescence intensity signal of control-silenced cells was 28.42± 1.06 A.U./μm^2^; Fig. 6A,Bc).

### TMEM170A interacts with RTN4 and the two proteins have antagonistic effects in ER and nuclear envelope morphogenesis

**Fig. 7.**
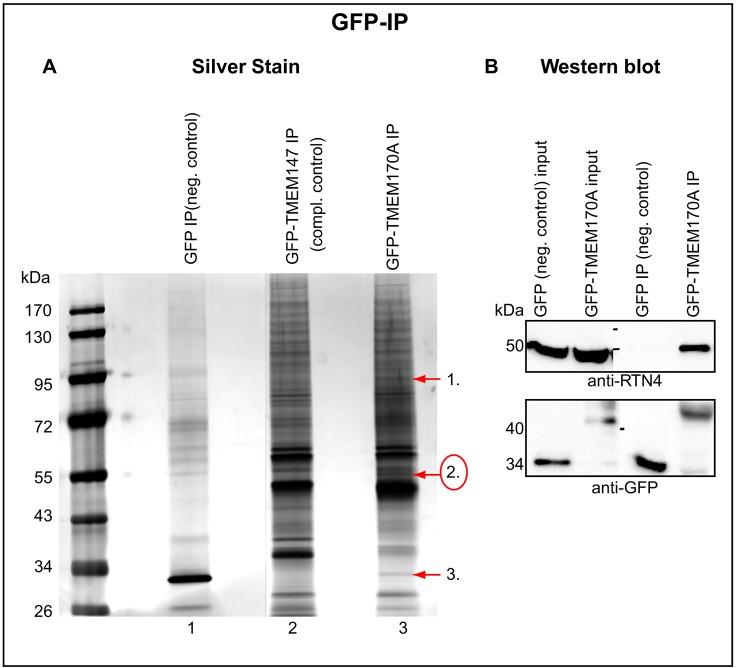
**TMEM170A interacts with RTN4.** (A) Silver-stained SDS-PAGE gel showing GFP (line 1), GFP–TMEM147 (line 2) control IPs and TMEM170A–GFP IP (line 3) with the use of GFP-Trap_A beads. Three bands, uniquely present in TMEM170A–GFP IP (arrows), were isolated and identified by liquid chromatography coupled with tandem mass spectrometry (Table S1). Band 2 (circled in red) was RTN4. (B) The anti-GFP IP was repeated and the interaction between TMEM170A and RTN4 was confirmed by western blot, using anti-GFP and anti-RTN4/NogoA+B antibodies. ‘Input’ corresponds to 1/40 volume of the lysate used for the reaction and ‘IP’ is 1/2 of the bound fraction, i.e. the protein complexes captured on the beads.

To investigate the mechanism through which TMEM170A exerts its effects on nuclear envelope and ER structure, we sought to identify its interacting partners by co-immunoprecipitation (IP). In brief, protein extracts from HeLa K cells stably expressing TMEM170A–GFP, GFP only (as negative control), or TMEM147–GFP (a control ER transmembrane protein for comparison) were incubated with GFP-Trap_A beads. Bound proteins were analyzed by SDS-PAGE electrophoresis, followed by silver staining (Fig. 7A). Three protein bands that were unique to the TMEM170A–GFP IP sample were identified by mass spectrometric analysis (Table S1). Band 2 was found to correspond to the RTN4 protein. The IP was repeated and the interaction between TMEM170A and RTN4 was also detected by western blot (Fig. 7B). Initial analysis showed that the 50 N-terminal and seven C-terminal amino acids of TMEM170A are not required for interaction with RTN4 (Fig. S3).

RTN4 was a particularly pertinent and promising candidate as it belongs to the reticulon family, which, together with the DP1/Yop1p family of proteins, is involved in shaping ER tubules, nuclear envelope formation and growth and NPC assembly (Voelz et al., 2006; Kiseleva et al., 2007; Anderson and Hetzer, 2008; Hu et al., 2008; Shibata et al., 2008, 2010; Dawson et al., 2009).

In order to understand the *in vivo* significance of the interaction between TMEM170A and RTN4, we compared the effects of single TMEM170A, single RTN4 or double TMEM170A plus RTN4 RNAi in HeLa K cells. We established conditions for efficient single and double silencing (Fig. S4A,B) and analyzed their effects on ER structure, NPC formation and nuclear envelope organization.

**Fig. 8.**
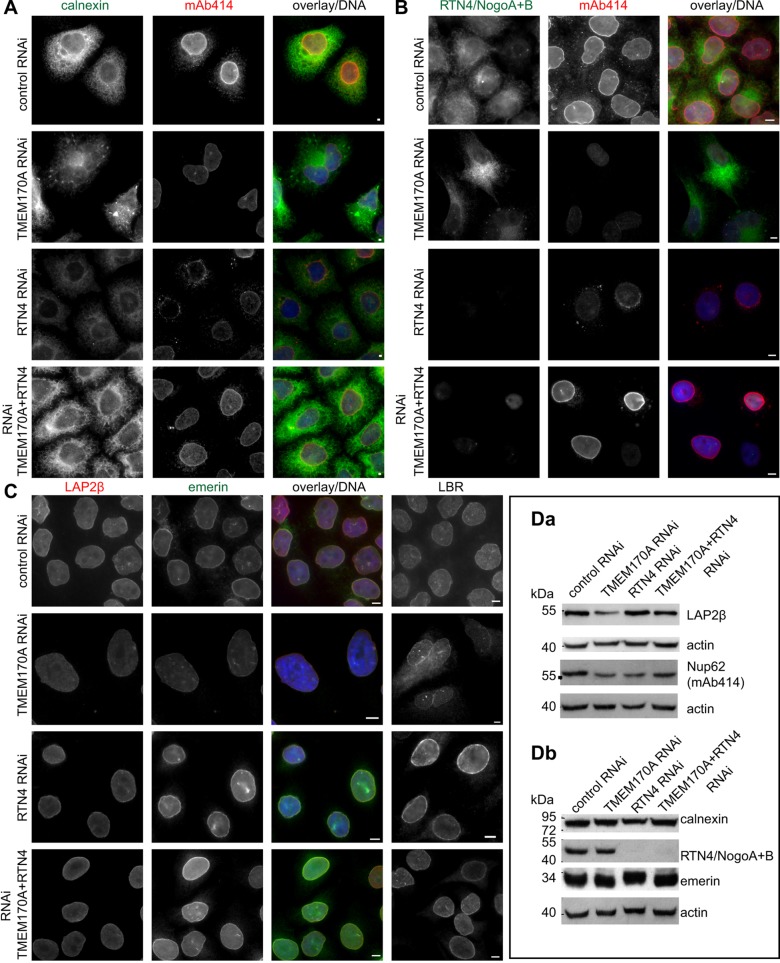
**Double TMEM170A plus RTN4 silencing restores the phenotypes caused either by single TMEM170A- or RTN4-silencing in HeLa K cells.** (A,B) Comparison of ER structure in cells silenced with control, single TMEM170A, RTN4 and double TMEM170A plus RTN4 RNAi, stained with anti-calnexin or anti-RTN4 (green) and mAb414 (red) showing that double silencing mostly reverses aberrant ER morphology and reduced nuclear rim signal induced by single TMEM170A silencing. Double TMEM170A- plus RTN4-silenced cells showed no altered phenotype, resembling control cells (upper row). (C) Equivalent experiment as in A,B, with cells stained for LAP2β (red) and emerin (green) or LBR (white). Single TMEM170A-silenced cells typically displayed reduced nuclear rim LAP2β or emerin signal and LBR was mislocalized to the ER. Single RTN4-silenced cells showed no phenotype but the double TMEM170A- plus RTN4-silenced cells exhibited restoration of LAP2β, emerin and LBR proteins to the nuclear envelope rim, as in controls. Nuclei in blue. Scale bars: 10 μm. (D) Western blot analysis of samples silenced with control, single TMEM170A, RTN4, or double TMEM170A plus RTN4 RNAi. Simultaneous TMEM170A plus RTN4 silencing restored to some extent the protein levels of nucleoporin Nup62 and LAP2β, compared with single TMEM170A or RTN4 silencing (Da). Single TMEM170A or RTN4 silencing and double TMEM170A plus RTN4 silencing have no effect on calnexin and emerin protein levels (Db).

As before, in single TMEM170A-silenced cells, ER structure was altered and exhibited enhanced aggregation (Fig. 8A; also Fig. 2A–D). No discernible ER organization phenotype was observed upon single RTN4 silencing (Fig. 8A), in agreement with previous studies documenting that all reticulon members must be co-depleted in order to observe ER sheet proliferation (Voeltz et al., 2006; Anderson and Hetzer, 2008). However, double TMEM170A plus RTN4 silencing led to typical ER organization, similar to that observed in negative controls (Fig. 8A, compare upper and bottom panels).

We then investigated whether double silencing also reverses the increased nuclear surface area caused by single TMEM170A depletion. Single TMEM170A-silenced cells showed an increase of their nuclear surface area to 145.68±4.82% of control cells (622.21±6.87 μm^2^ in controls vs 906.58±36.52 μm^2^ in TMEM170A-silenced cells, *P=*0.00019; Fig. S4C1). Single RTN4-silenced cells did not show a reduction of their nuclear surface area (568.37±38.57 μm^2^ in RTN4-silenced cells vs 622.21±6.87 μm^2^ in controls; Fig. S4C). We found that simultaneous co-silencing of TMEM170A plus RTN4 restored nuclear size to similar levels observed in negative control cells (679.87±34.37 μm^2^ in TMEM170A- plus RTN4-silenced cells vs 622.21±6.87 μm^2^ in controls; Fig. S4C1).

In addition, we investigated whether this double silencing also reverses the increased nuclear volume caused by single TMEM170A depletion. Single TMEM170A-silenced cells showed an increase of their nuclear volume to 137.38±1.13% of control cells (867.91± 16.51 μm^3^ in controls vs 1192.4±28.39 μm^3^ in TMEM170A-silenced cells, *P=*6.84×10^−5^; Fig. S4C2). Single RTN4-silenced cells showed a reduction of nuclear volume to 73.48±4.27% of control cells (638.11±46.04 μm^3^ in RTN4-silenced cells vs 867.91±16.51 μm^3^ in controls, *P=*0.001; Fig. S4C); this agrees with a previous study showing that addition of anti-RTN4A antibody to nuclear assembly reactions inhibited nuclear envelope growth (Kiseleva et al., 2007). Here, simultaneous co-silencing of TMEM170A plus RTN4 restored nuclear size to similar levels as observed in negative control cells (931.03±74.78 μm^3^ in TMEM170A- plus RTN4-silenced cells vs 867.91±16.51 μm^3^ in controls; Fig. S4C2).

We also examined whether double silencing reverses the reduction in NPC formation, caused by single TMEM170A depletion. Single TMEM170A-silenced cells showed reduced nuclear rim signal relative to controls, using the mAb414 antibody as a marker (Fig. 8A,B; also Fig. 4A). Single RTN4-silenced cells also showed reduced nuclear rim signal and nucleoporins mislocalizing to the cytoplasm compared with controls (Fig. 8A,B). These observations were in line with findings that an rtn1Δyop1Δ yeast mutant exhibited NPC clusters on the nuclear envelope (Dawson et al., 2009) and the *in vitro* inhibition of *de novo* NPC formation in *Xenopus laevis* egg extract upon addition of anti-RTN4 antibody (Dawson et al., 2009). Again, as in the case of the ER, simultaneous co-silencing of TMEM170A plus RTN4 resulted in NPC phenotypes reminiscent of control cells (Fig. 8B). Furthermore, single TMEM170A-silenced cells showed a reduction of NPC density in silenced cells to 69.58±12.70% of control cells stained for ELYS (28.42±1.06 A.U./μm^2^ in controls vs 19.85±4.15 A.U./μm^2^ in TMEM170A-silenced cells, *P=*0.025) and a reduction of NPC density in TMEM170A-silenced cells to 58.72±22.16% of control cells stained with mAb414 (24.93±2.96 A.U./μm^2^ in controls vs 14.26±3.76 A.U./μm^2^ in TMEM170A-silenced cells, *P=*0.018; Fig. S4C3). Single RTN4-silenced cells also showed a reduction of NPC density in silenced cells to 68.32±4.48% compared with control cells, stained for ELYS (28.42±1.06 A.U./μm^2^ in controls vs 19.45±1.96 A.U./μm^2^ in RTN4-silenced cells, *P=*0.002), and a reduction of NPC density in silenced cells to 65.69±17.12% compared with control cells, stained with mAb414 (24.93±2.96 A.U./μm^2^ in controls vs 16.09±2.59 A.U./μm^2^ in RTN4-silenced cells, *P=*0.017; Fig. S4C3). In this case, simultaneous co-silencing of TMEM170A plus RTN4 restored the NPC density to similar levels as observed in negative control cells (For ELYS: 25.6±2.21 A.U./μm^2^ in TMEM170A- plus RTN4-silenced cells vs 28.42±1.06 A.U./μm^2^ in controls; for mAb414: 20.52±2.32 A.U./μm^2^ in TMEM170A- plus RTN4-silenced cells vs 24.93±2.96 A.U./μm^2^ in controls; Fig. S4C3).

Finally, we investigated whether double silencing also restores the level of INM proteins at the rim. Indeed, in double TMEM170A- plus RTN4-silenced cells, emerin and LBR were now correctly localized to the nuclear envelope rim, in contrast to single TMEM170A-silenced cells where emerin rim staining is reduced and LBR is partly mislocalized to the ER (Fig. 8C). Upon single RTN4 silencing, no discernible INM-protein-related phenotype was observed (Fig. 8C). In addition, simultaneous co-silencing of TMEM170A plus RTN4 also mostly restored LAP2β protein on the nuclear envelope rim, compared with single TMEM170A-silenced cells where LAP2β protein showed, as before, reduced nuclear rim signal intensity (Fig. 8C).

Consistent with the above results, western blot analysis showed that double TMEM170A plus RTN4 silencing restored the levels of nucleoporins and INM proteins that were reduced by single TMEM170A or RTN4 silencing. For example, single silencing of either TMEM170A or RTN4 causes a modest reduction the protein levels of Nup62 (as detected by mAb414), which is then mostly restored by double TMEM170A plus RTN4 silencing (Fig. 8Da). Additionally, single TMEM170A silencing causes a reduction in LAP2β protein levels but this reduction was restored by double silencing (Fig. 8Da). Calnexin or emerin protein levels did not seem affected by any of the silencing regimes (Fig. 8Db).

Taking into consideration the rescue of ER-, NPC- and nuclear-envelope-related phenotypes by simultaneous co-silencing of TMEM170A and RTN4, we conclude that the two proteins have antagonistic effects on all three processes: ER membrane organization, nuclear envelope organization and NPC formation.

## DISCUSSION

Deciphering the mechanisms of membrane morphogenesis in the ER remains a major challenge in cell biology. Although the ER is clearly an extremely important cellular compartment as the site of protein synthesis, processing and assembly as well as a membrane supplier and membrane flow regulator, its sheer size, accounting for about 50% of the total membrane volume in the cell, and its structural complexity, encompassing the nuclear envelope and tubular and sheet forms, make the task both technically and conceptually difficult.

Consequently, and despite the considerable recent advances outlined in the Introduction, our overall understanding is still incomplete and sketchy. In this study we identified and characterized TMEM170A, a transmembrane domain protein that localizes in all parts of the ER (nuclear envelope, tubular ER and ER sheets). Our *in vivo* results build a strong case for TMEM170A being the first example of an ER protein functioning specifically to promote ER sheet formation. Downregulation of TMEM170A by siRNA alters ER shape and, as revealed by TEM and 3D electron tomography, this is caused by the formation of excessive tubular ER. By contrast, overexpression of TMEM170A was found to promote ER sheet formation. The combination of these results indicates that the cellular levels of TMEM170A can influence the ratio of tubular ER to ER sheets, supporting the notion that TMEM170A promotes ER sheet formation at the expense of ER tubules.

How does TMEM170 function? The mechanism through which ER sheets are formed is far from clear. The reticulon and DP1/Yop1p family members, which are responsible for shaping tubular ER, have also been implicated in shaping ER sheets (Shibata et al., 2010). These proteins, localized on the edge of ER sheets, create a positive membrane curvature, thus bending the membrane surface, bringing two membrane sheets in close proximity and stabilizing them, possibly through oligomerization into scaffold structures (Shibata et al., 2010). In addition, several rough ER proteins, for example proteins of the translocon complex and coiled-coil transmembrane proteins such as CLIMP-63, kinectin and p180, all of which specifically localize on ER sheets, have a role in ER sheet morphogenesis. CLIMP-63 has its coiled-coil domain facing the luminal side of the ER membrane and forms bridges that keep the luminal width of the ER sheets constant (Klopfenstein et al., 2001). Overexpression of CLIMP-63 induces ER sheet formation whereas its silencing does not abolish the presence of ER sheets but, instead, causes a dramatic decrease of sheet luminal thickness (Shibata et al., 2010). Single or double silencing of kinectin and p180, which have their coiled-coil domain facing the cytoplasm and possibly contribute to the flatness of the ER sheets, does not influence ER structure (Shibata et al., 2010). It appears, therefore, that although all these proteins are important in some aspect of ER sheet morphology they are not essential to promote ER sheet formation (Shibata et al., 2010).

To start unraveling the mechanism through which TMEM170A exerts its action in sheet formation, we searched for its interacting proteins partners. Interestingly, we identified RTN4, a well-known tubular-ER-shaping protein, whose overexpression results in ER tubule proliferation (Voeltz et al., 2006). It was intriguing that simultaneous double silencing of TMEM170A (a sheet-promoting protein) and RTN4 (a tubule-promoting protein) was found to restore the altered ER structure that we had observed with single TMEM170A silencing. This finding, indicating that TMEM170A and RTN4 act antagonistically in ER membrane formation, is not unprecedented; co-overexpression of sheet-lumen-bridging protein CLIMP-63 and RTN4 also resulted in normal ER structure (Shibata et al., 2010). In addition, TMEM33, another transmembrane ER protein, was recently shown to interact with reticulon proteins (RTN1A, RTN2B, RTN3C and RTN4C) and TMEM33 overexpression suppressed the excess tubulation of ER induced by RTN4C overexpression (Urade et al., 2014). These results suggest that a number of tubular- and sheet-forming ER proteins act to balance the ratio of tubular ER to ER sheets during ER morphogenesis and cell growth. Whether TMEM170A actively promotes ER sheet formation, or whether it instead acts by inhibiting factors that promote ER tubule formation, is a challenging future question.

The role of TMEM170A does not seem limited to the promotion of ER sheet formation. The nuclear envelope, which is a distinct part of the ER and, itself, a double membrane sheet, is also affected by TMEM170A. Silencing of TMEM170A causes nuclear expansion whereas its overexpression inhibits nuclear growth. This result was specific as in rescue experiments cells had normal nuclear size. Taken together, these *in vivo* results indicate that overproliferation of cytoplasmic ER sheets can inhibit nuclear envelope expansion. It has been shown *in vitro* that membrane feeding from ER to the ONM for nuclear envelope expansion occurs through tubular ER connections and that disturbing peripheral tubular ER connections with the nuclear envelope affects nuclear envelope expansion (Anderson and Hetzer, 2007). It is possible that the increased nuclear size in TMEM170A-silenced cells is caused by increased delivery of membranes from tubular ER connections to the nuclear envelope. The reticulon family members have also been implicated in nuclear envelope expansion, although published results on this point to date appear contradictory. Addition of an RTN4-neutralising antibody in an *in vitro* assay inhibited nuclear envelope growth (Kiseleva et al., 2007) whereas, in another study, overexpression of RTN4 in cells inhibited nuclear envelope growth (Anderson and Hetzer, 2008). In the second study, it was also shown that simultaneous silencing of the RTN1, RTN3 and RTN4 reticulon proteins did not accelerate nuclear envelope expansion (Anderson and Hetzer, 2008). In this work, we showed that double silencing of TMEM170A plus RTN4, in addition to restoring normal ER structure, also restored normal nuclear size, indicating that the correct balance of ER-shaping proteins also influences nuclear envelope expansion, most probably by affecting the correct connection of nuclear envelope to the peripheral ER and even the mechanism through which membranes are transferred from ER to the nuclear envelope.

Our results also implicate TMEM170A in NPC formation. TMEM170A-silenced cells showed a dramatic decrease in NPC density and, in addition, cellular protein levels of several nucleoporins were markedly reduced. Co-silencing of TMEM170A plus RTN4 restored the NPC phenotypes and nucleoporin cellular protein levels, indicating that TMEM170A and RTN4 act antagonistically in NPC assembly. How exactly RTN4 and TMEM170A are implicated in NPC formation is an important question that needs to be addressed in the future.

Finally, we have shown that TMEM170A affects the accumulation and localization of INM proteins. In TMEM170A-silenced cells, the INM proteins we studied are either mislocalized to the ER or accumulate to lower levels at the nuclear rim. INM proteins are synthesized in ER, diffuse to the ONM and then either diffuse or are transported to the INM via the NPC (Mattaj, 2004). NPCs control the movement of proteins from ONM to INM and certain nucleoporins, such as Nup188 and gp210, can act as ‘gatekeepers’ controlling protein flux to the INM (Ohba et al., 2004; Theerthagiri et al., 2010). Again, we noted that TMEM170A and RTN4 have antagonistic effects on these phenotypes, suggesting that, although direct effects cannot be ruled out, the phenotypes might be secondary consequences of changes in the ER membrane system or in NPC formation.

In summary, TMEM170A is a transmembrane protein of the ER that promotes ER sheet formation at the expense of ER tubules and that interacts with RTN4. Taking into consideration the rescue of ER-, NPC- and nuclear-envelope-related phenotypes by simultaneous co-silencing of TMEM170A and RTN4, we concluded that the activities of TMEM170A and RTN4 are in a functional ‘tug of war’, where TMEM170A appears to shift the balance towards ER sheet formation whereas RTN4 promotes tubular ER production. Our results strengthen the argument that, for correct ER morphogenesis, a balance between tubular and ER-sheet-promoting proteins is necessary and suggest that it should be possible to regulate the ratio of tubular ER to ER sheets depending on cellular conditions.

## MATERIALS AND METHODS

### RT-PCR and quantitative real-time PCR

RNA was extracted from HeLa K cells with the RNeasy purification kit (Qiagen); 1 μg used for reverse transcription using Protoscript Reverse Transcription Kit with dT23VN primer (New England Biolabs).

For relative quantitation of mRNA in silencing experiments, real time RT-PCR was conducted using the LightCycler system (Roche) with FastStar DNA Master SYB Green I reagent (Roche) and specific primers for TMEM170A (primer set 1) and mammaglobin-2 (MGB2; primer set 2 as a standard; Table S2).

### Plasmid vectors

For mammalian expression, the full-length ORF of TMEM170A was amplified from HeLa K with primer set 3 (Table S2), transferred as an *Xho*I/*Eco*RI fragment into pEGFP-N1 (Clontech) and subcloned as a *Bgl*II/*Kpn*I fragment into pFLAG-CMV-2 (Sigma-Aldrich). For bacterial expression, full-length TMEM170A was amplified with primer set 4 (Table S2) transferred as an *Eco*RI/*Xho*I fragment into pET28a (Novagen) and subcloned as a *Bam*HI/*Xho*I fragment into mammalian expression vector pSVmyc1.0 (Santama et al., 1998). The ORF of TMEM170A mutants, lacking the first 49 N-terminal amino acids (TMEM170Am_50-144) or the last C-terminal 7 amino acids (TMEM170Am_1-137), was amplified with primer sets 5 and 6, respectively (Table S2) and transferred as an *Xho*I/*Eco*RI fragment into pEGFP-N1 (Clontech).

### Cell culture

HeLa Kyoto (HeLa K) and human osteosarcoma U2OS cells were cultured in DMEM, supplemented with 10% v/v fetal bovine serum (FBS), 2 mM glutamine and 50 U/ml of penicillin/streptomycin, at 37°C in 5% CO_2_. HeLa K TMEM170A–GFP cell line was generated by transfection of pEGFPN1-TMEM170A and selected in the same medium, supplemented with 0.5 mg/ml G418 (Invitrogen).

### Plasmid transfections and RNA interference

Cells were transiently transfected with 5 μg plasmid DNA, using Lipofectamine 2000 (Invitrogen) and according to the manufacturer's instructions.

Transfections for RNAi experiments were performed using INTERFERin (Polyplus Transfection) and annealed siRNA oligos, specific for TMEM170A or RTN4 or negative control (Ambion Inc. USA and MWG, Germany), at a final concentration of 20 nM. For double silencing, a cocktail of two sets of siRNAs (TMEM10A and RTN4) was used (10 nM each). All siRNA oligos are listed in Table S3. Cells were harvested for immunofluorescence, real-time RT-PCR and western blot analysis after 72 h (48 h with oligo set 3).

### Antibodies

All primary and secondary antibodies are listed in Table S4.

### Immunofluorescence

Cells grown on coverslips were fixed in 4% w/v paraformaldehyde in PBS (10 min) and permeabilized (5 min) with 0.5% v/v Triton X-100 in PBS or fixed and permeabilized in methanol (10 min) at −20°C. All cells were quenched (15 min) with 50 mM NH_4_Cl in PBS, blocked with 2% w/v BSA, 2% v/v FCS, 0.2% v/v fish skin gelatin in PBS (blocking mix) (1 h) and incubated with primary and secondary antibodies in PBS, 5% v/v blocking mix (1 h).

For the experiments in Fig. 1B, HeLa K cells expressing TMEM170A–GFP protein were fixed with 4% w/v paraformaldehyde, permeabilized with 30 μg/ml digitonin in PBS on ice (5 min) or 30 μg/ml digitonin, 0.5% v/v Triton X-100 in PBS on ice (5 min), washed with PBS and processed for immunofluorescence.

Immunofluorescent preparations were analyzed on a Zeiss LSM 510 META or Zeiss LSM 710 Αxiovert confocal microscope using a 63× Plan-Neofluar 1.4 NA oil immersion objective lens or on a Zeiss Axiovert 200M inverted fluorescence microscope using Zeiss Apochromat 63×1.4 NA oil lens, or Leica SP8 CSU using a 63×1.4 NA oil lens. Images were analyzed with Zeiss LSM, Zen or Axiovision 4.2 software or Image J (NIH) and processed using Adobe Photoshop.

### Quantitation of nuclear volume, nuclear surface area and NPC density

To determine nuclear volume, nuclear surface area and NPC density, cells were immunostained with anti-MEL28/ELYS and mAb414 antibodies, and Hoechst. For each nucleus, the volume, surface area and mean fluorescence intensity were measured from >20 confocal *z*-series, spaced 0.2 μm apart and spanning the entire nucleus, using Imaris 8.1 (Bitplane). Data for each condition tested were collected from three independent experiments and combined for statistical analysis.

### Electron microscopy

Cells were processed for electron microscopy according to Platani et al. (2009). Serial sections were cut either 60 nm or 250 nm thick and placed on copper palladium slot grids coated with 1% Formvar (Serva, Germany). Thin sections were imaged with a CM120 Phillips electron microscope. Electron tomography was done on thick sections with a Technai F30 300 kV microscope (FEI Company). Serial sections were reconstructed and tomograms were joined using IMOD (Kremer et al., 1996). HeLa K FLAG–TMEM170A-expressing cells in Fig. 3Bc,d were seeded onto carbon-coated sapphire discs 0.15 mm thick (M. Wohlwend GmbH, Switzerland) and high-pressure frozen on a BalTec HPM010 freezing machine (ABRA Fluid, Switzerland). Cells were then freeze-substituted in 1% OsO4 and 0.1% uranyl acetate in acetone and embedded into EPON (Serva, Germany). Sections (60 nm) were placed on copper palladium slot grids, coated with 1% Formvar, and imaged with a CM120 Phillips electron microscope.

### Immunoprecipitation and liquid chromatography coupled with tandem mass spectrometry analysis

Ten square 25-cm dishes (each 500 cm^2^) of HeLa K TMEM170A–GFP or TMEM147–GFP cell lines or HeLa K GFP cell line (negative control) were lysed in 2 ml lysis buffer [20 mM Tris pH 7.5, 150 mM NaCl, 1% v/v NP40 and 1 tablet/50 ml of Complete protease inhibitor cocktail (Roche)]. Each of the three extracts was incubated with a 20 μl slurry of GFP-Trap_A beads (Chromotek) for 2 h at 4°C. After binding, beads were extensively washed in lysis buffer and bound proteins were eluted from beads using 50 μl SDS-PAGE sample buffer. Boiled samples were analyzed by SDS-PAGE electrophoresis, followed by silver staining. Bands unique to the TMEM170A–GFP samples were cut out, trypsin-digested in-gel and eluted, and tryptic peptides were separated and analyzed by liquid chromatography coupled with tandem mass spectrometry (Orbitrap Velos, Thermo Scientific) at the EMBL Proteomics Core Facility.

### Computational analysis

Protein alignment (Fig. S1) was generated using Clustal Omega (http://www.ebi.ac.uk/Tools/msa/clustalo/).

Transmembrane domains of TMEM170A were predicted with TMPRED software (http://www.ch.embnet.org/software/TMPRED_form.html).

### Statistical analysis

Numerical data were analyzed and graphically represented using Microsoft Excel. Statistical significance was determined by paired or homoschedastic, two-tailed Student's *t*-test.
